# Association of Combined Effect of Metals Exposure and Behavioral Factors on Depressive Symptoms in Women

**DOI:** 10.3390/toxics12120879

**Published:** 2024-12-02

**Authors:** Olamide Ogundare, Emmanuel Obeng-Gyasi

**Affiliations:** 1 Department of Built Environment, North Carolina A&T State University, Greensboro, NC 27411, USA; 2 Environmental Health and Disease Laboratory, North Carolina A&T State University, Greensboro, NC 27411, USA

**Keywords:** depressive symptoms, metals exposure, behavioral factors, Bayesian kernel machine regression (BKMR), posterior inclusion probability (PIP)

## Abstract

This study investigates the combined effects of environmental pollutants (lead, cadmium, total mercury) and behavioral factors (alcohol consumption, smoking) on depressive symptoms in women. Data from the National Health and Nutrition Examination Survey (NHANES) 2017–2018 cycle, specifically exposure levels of heavy metals in blood samples, were used in this study. The analysis of these data included the application of descriptive statistics, linear regression, and Bayesian Kernel Machine Regression (BKMR) to explore associations between environmental exposures, behavioral factors, and depression. The PHQ-9, a well-validated tool that assesses nine items for depressive symptoms, was used to evaluate depression severity over the prior two weeks on a 0–3 scale, with total scores ranging from 0 to 27. Exposure levels of heavy metals were measured in blood samples. BKMR was used to estimate the exposure–response relationship, while posterior inclusion probability (PIP) in BKMR was used to quantify the likelihood that a given exposure was included in the model, reflecting its relative importance in explaining the outcome (depression) within the context of other predictors in the mixture. A descriptive analysis showed mean total levels of lead, cadmium, and total mercury at 1.21 µg/dL, 1.47 µg/L, and 0.80 µg/L, respectively, with a mean PHQ-9 score of 5.94, which corresponds to mild depressive symptoms based on the PHQ-9 scoring. Linear regression indicated positive associations between depression and lead as well as cadmium, while total mercury had a negative association. Alcohol and smoking were also positively associated with depression. These findings were not significant, but limitations in linear regression prompted a BKMR analysis. BKMR posterior inclusion probability (PIP) analysis revealed alcohol and cadmium as significant contributors to depressive symptoms, with cadmium (PIP = 0.447) and alcohol (PIP = 0.565) showing notable effects. Univariate and bivariate analyses revealed lead and total mercury’s strong relationship with depression, with cadmium showing a complex pattern in the bivariate analysis. A cumulative exposure analysis of all metals and behavioral factors concurrently demonstrated that higher quantile levels of combined exposures were associated with an increased risk of depression. Finally, a single variable-effects analysis in BKMR revealed lead, cadmium, and alcohol had a stronger impact on depression. Overall, the study findings suggest that from exposure to lead, cadmium, mercury, alcohol, and smoking, cadmium and alcohol consumption emerge as key contributors to depressive symptoms. These results highlight the need to address both environmental and lifestyle choices in efforts to mitigate depression.

## 1. Introduction

Depression is a significant global health concern, ranking as one of the leading causes of disability worldwide [1]. An estimated 3.8% of the global population experiences depression, including 5% of adults (4% of men and 6% of women) and 5.7% of adults over the age of 60. Approximately 280 million people worldwide are affected by depression [2,3]. It poses a serious threat to the quality of life, often leading to diminished social functioning, inability to work, and severe emotional suffering. Depression is a major contributor to suicide deaths, a connection that is often underrepresented in global assessments of disease burden [4]. Depression typically manifests through a range of symptoms, including persistent sadness, feelings of hopelessness, and fatigue. Individuals may experience changes in sleep patterns, appetite, and a withdrawal from social interactions [5]. While the disorder affects people of all ages, its prevalence can vary greatly depending on the population studied. For instance, among older adults, the prevalence of depression ranges from 1% to 49% and is influenced by factors such as physical health and social isolation [6]. In the United States, depression rates among young people have shown a significant rise in recent years, increasing from 3.1% in 2016 to 4.0% in 2020—an approximately 30% increase over a five-year period. This trend is particularly pronounced among females, emphasizing the urgent need for targeted interventions [7]. Adolescence is a particularly critical period for the onset of depression, as untreated depression during this time can persist into adulthood, compounding the impact on long-term mental health [8].

Women are disproportionately affected by depression owing to a range of biological, social, and environmental factors. Globally, the prevalence of depression is higher in women than in men [9]. The sex-based differences in depression may be partly attributed to estrogen, a major female sex hormone [10]. Hormonal fluctuations during different life stages—such as menstruation, pregnancy, postpartum, and menopause—play a significant role in increasing depression risk among women. Additionally, the burden of caregiving responsibilities, experiences of gender-based violence, and societal pressures contribute to the higher prevalence of depression in women compared to men. Beyond these social stressors, environmental exposures also play a crucial role. For instance, research has shown that women are more vulnerable to the neurotoxic effects of heavy metals, such as lead (Pb) and cadmium (Cd) [11,12]. There is growing evidence that metal exposure is more strongly associated with mood disorders in women, likely due to physiological and hormonal differences between the sexes [13]. This highlights the importance of addressing sex-specific factors in developing effective prevention and treatment strategies for depression in women.

The Patient Health Questionnaire-9 (PHQ-9) is a widely utilized screening tool to assess depression severity [14]. It consists of nine questions that inquire about depressive symptoms over the past two weeks, such as changes in sleep, appetite, and mood. The PHQ-9 has been validated as a reliable and sensitive instrument for diagnosing depression [15], making it an essential tool for clinicians and researchers alike.

In addition to biological and social factors, behavioral factors such as alcohol consumption and smoking are closely linked to depression. Alcohol abuse can exacerbate depressive symptoms, often creating a vicious cycle where individuals self-medicate with alcohol, further worsening their condition. Smoking is also highly prevalent among individuals with mental illness, complicating the treatment of depression and increasing the risk of other health problems [16]. In women, the rising rates of alcohol misuse are particularly concerning, as they tend to experience harmful health and behavioral effects from drinking sooner or at lower levels of alcohol consumption compared to men [17]. There are multiple reasons why this may occur. Women are typically smaller than men and have less total body water and more body fat. Consequently, alcohol becomes more concentrated in a woman’s body, causing blood alcohol levels to rise more quickly and remain elevated longer compared to men [18].

The risk factors for women who smoke are higher in comparison with their male counterparts. Smoking has been identified as an independent risk factor for ectopic pregnancy, as it disrupts fallopian tube function and ovulation. This results in a two-fold higher risk of the ectopic implantation of a fertilized egg in women who smoke compared to those who do not [19]. Women who smoke are three times more likely to experience delayed conception (taking over a year to conceive) compared to non-smokers, due to an early decline in gonadotropin levels and follicular atresia [20]. A study has shown that women who begin smoking in adolescence and those with a higher tobacco consumption are at an increased risk of premenstrual syndrome and experience more irregular and shorter menstrual cycles compared to non-smokers [21]. 

Emerging evidence has also pointed to environmental toxicants, particularly heavy metals, as contributing factors to depression in women [22]. Heavy metals such as cadmium, lead, and total mercury are known neurotoxicants that have been associated with a range of cognitive and mood disorders [23]. Exposure pathways to cadmium include smoking, soldering, and contact with cadmium-containing products such as nickel-chromium batteries, jewelry, toys, and electronic devices [24]. Lead exposure, for instance, has been linked to mood disturbances and anxiety, even at low levels of exposure [25]. Total mercury exposure, commonly through the consumption of contaminated seafood, poses risks to mental health, though it is sometimes mitigated by the intake of protective nutrients like the omega-3 fatty acids found in fish [26]. Despite extensive research on depression, there is still a lack of studies that examine the combined effects of biological, social, behavioral, and environmental factors. Bridging this gap in the research could provide critical insights into the complex and multifactorial nature of depression, ultimately improving prevention and treatment approaches.

## 2. Materials and Methods

### 2.1. Study Design

This study analyzed data from the 2017–2018 National Health and Nutrition Examination Survey (NHANES), a cross-sectional study designed to assess the health and nutritional status of the U.S. non-institutionalized population, to examine the effects of environmental and behavioral factors on depressive symptoms among women. The survey, conducted by the U.S. Centers for Disease Control and Prevention (CDC), employs a multi-stage, stratified sampling design and collects data in two-year cycles. Participants from across all 50 states and Washington, D.C., provide informed consent before undergoing physical examinations and structured interviews. Blood samples are collected for laboratory analysis, and demographic data—such as age, sex, and race/ethnicity—are gathered using a Computer-Assisted Personal Interview (CAPI) system to enhance accuracy. The NHANES protocols were reviewed and approved by the Institutional Review Board at the National Center for Health Statistics (NCHS), a division of the CDC.

### 2.2. Variables and Covariates

The primary outcome variable in this study was depression, measured using the PHQ-9 questionnaire [27]. Predictor variables included behavioral factors such as smoking and alcohol consumption, as well as environmental exposure to heavy metals (lead, cadmium, and total mercury). The analysis also adjusted for covariates, including age, sex, race/ethnicity, and income, to account for potential confounding effects.

### 2.3. Measurement of Heavy Metals (Lead, Cadmium, and Total Mercury)

Levels of heavy metals in whole blood samples were quantified using inductively coupled plasma dynamic reaction cell mass spectrometry (ICP-DRC-MS). The assays were conducted by the CDC’s Division of Laboratory Sciences at the National Center for Environmental Health. Detailed procedures on quality control and assurance can be found in NHANES technical documentation [28]. The metals were measured, and the concentrations were expressed in micrograms per liter (µg/L) for cadmium and total mercury and (µg/dL) for lead.

### 2.4. Alcohol and Smoking Assessment

Alcohol consumption data were collected via the NHANES Alcohol Use Questionnaire, which captures information on both lifetime and current drinking patterns. Smoking status was determined through self-reported data on cigarette use, with a focus on whether individuals were current smokers. The data analysis for both variables was based on frequency of use [29,30].

### 2.5. Depressive Symptoms

In this NHANES study, participants completed the PHQ-9 survey during in-person interviews conducted at mobile examination centers (MECs). Of the total participants, 153 individuals met the inclusion criteria for our analysis pool, which required complete data for the PHQ-9 and all other relevant environmental and behavioral variables.

The PHQ-9 asked participants to report the frequency of depressive symptoms over the prior two weeks, using a 0 to 3 scale. The symptoms assessed included the following: (a) loss of interest or pleasure in activities (anhedonia), (b) persistent sadness, (c) disturbances in sleep, (d) fatigue, (e) changes in appetite, (f) feelings of low self-worth, (g) trouble concentrating, (h) psychomotor disturbances, and (i) thoughts of self-harm. Total PHQ-9 scores range from 0 to 27, with a score of 10 or higher indicating clinically significant depression [15]. The PHQ-9 is a well-validated tool for assessing depression severity, with scores corresponding to minimal (1–4), mild (5–9), moderate (10–14), moderately severe (15–19), and severe depression (20–27) [27,31]. The instrument demonstrates high internal consistency, sensitivity, and specificity for detecting major depressive disorder [31,32].

### 2.6. Statistical Analysis

#### 2.6.1. Descriptive Statistics and Regression Analysis

Descriptive statistics were used to summarize key demographic characteristics, such as age, sex, and race/ethnicity. Linear regression models were employed to explore the association between depression (the outcome variable) and the predictor variables (behavioral factors and environmental exposures). The analysis was adjusted for covariates to control for confounding effects, and complete data were utilized for the modeling.

#### 2.6.2. Bayesian Kernel Machine Regression (BKMR)

Bayesian Kernel Machine Regression (BKMR) was utilized to examine the combined effects of multiple environmental exposures on depression. BKMR is a flexible regression technique that accounts for complex, non-linear relationships between multiple exposures and health outcomes. This approach allows for the detection of potential interactions between different contaminants that cannot be captured using traditional linear regression models. In the BKMR model, exposure–response relationships are modeled nonparametrically using Gaussian kernels, and the relationships between exposures and depression were estimated using Markov Chain Monte Carlo (MCMC) sampling. A total of 50,000 iterations were run to ensure parameter convergence. All statistical analyses were conducted in R software (version 4.2.3), with a significance level of 0.05 applied to non-Bayesian analyses. All analyses were adjusted for age, sex, ethnicity, and income.

The posterior inclusion probability (PIP) was calculated within the BKMR framework to quantify the relative importance of the variables lead, cadmium, total mercury, alcohol, and smoking. PIP represents the posterior probability that a particular variable contributes to the model, with higher values indicating greater importance. PIP values were derived from the posterior samples generated during MCMC iterations. Specifically, the PIP was calculated as the proportion of MCMC samples in which the inclusion indicator for a given variable was nonzero. This allowed for a robust assessment of the contribution of individual variables to the overall depression outcome, while accounting for potential interactions and collinearity between variables.

Group PIP and conditional PIP were also calculated to further investigate the influence of exposures on depression. Group PIP quantifies the combined importance of a predefined group of exposures by summing the inclusion probabilities of all exposures within the group. In this study, the groups were metals (lead, cadmium, and total mercury) and behavior variables (smoking and alcohol consumption). Group PIP values reflected the collective contribution of metals or behavior variables to depression. Conditional PIP, on the other hand, evaluates the importance of an individual exposure while conditioning this on the presence of other exposures in the mixture, isolating its unique contribution from potential confounding effects. For instance, conditional PIP allowed us to determine the independent effects of lead, cadmium, or smoking, while accounting for the influence of other exposures. Both group PIP and conditional PIP were calculated from the posterior samples generated during MCMC iterations, leveraging the flexibility of the BKMR framework to explore these relationships in detail. 

## 3. Results

### 3.1. Descriptive Statistics

Table 1 presents the descriptive statistics for the continuous variables included in the study. The sample consisted of 153 participants, with an average depression score (PHQ-9) of 5.94 (SD = 5.73), indicating that, on average, the study population falls within the mild depression category. Blood lead levels had a mean of 1.21 µg/dL (SD = 0.679), while cadmium levels averaged 1.47 µg/L (SD = 1.06). Total mercury levels in the blood were lower, with a mean of 0.80 µg/L (SD = 0.93). The mean age of participants was 48.03 years (SD = 14.91), reflecting a middle-aged study population.

Within this study, the largest group consisted of non-Hispanic White individuals, making up 49.67% of the sample. Non-Hispanic Black participants represented 27.45%, while 9.8% were Mexican American. Individuals from other Hispanic backgrounds comprised 3.27% of the sample, and non-Hispanic Asians accounted for 1.31%. The category labeled “Other Race”, which includes participants identifying with multiple races or other racial categories, made up 8.5% of the study population. This diverse racial/ethnic distribution highlights the study’s inclusion of various demographic groups, which is critical for exploring how environmental exposures and depression may vary across different populations.

### 3.2. Linear Regression

Table 2 displays the linear regression results examining the associations between depression and various predictors, including heavy metals and behavioral factors. Lead exposure showed a positive relationship with depression (β = 1.21), suggesting that higher lead levels are associated with increased depressive symptoms. Cadmium also had a positive association (β = 0.20), though the effect was smaller. In contrast, total mercury displayed a negative association with depression (β = −0.52). Additionally, alcohol use and smoking both showed positive relationships with depression, with alcohol (β = 0.14) and smoking (β = 0.01) slightly associated with higher depressive symptoms. None of these relationships were statistically significant, but the trends warranted further investigation using more sophisticated methods.

### 3.3. Spearman Correlation Analysis

**Figure 1:**
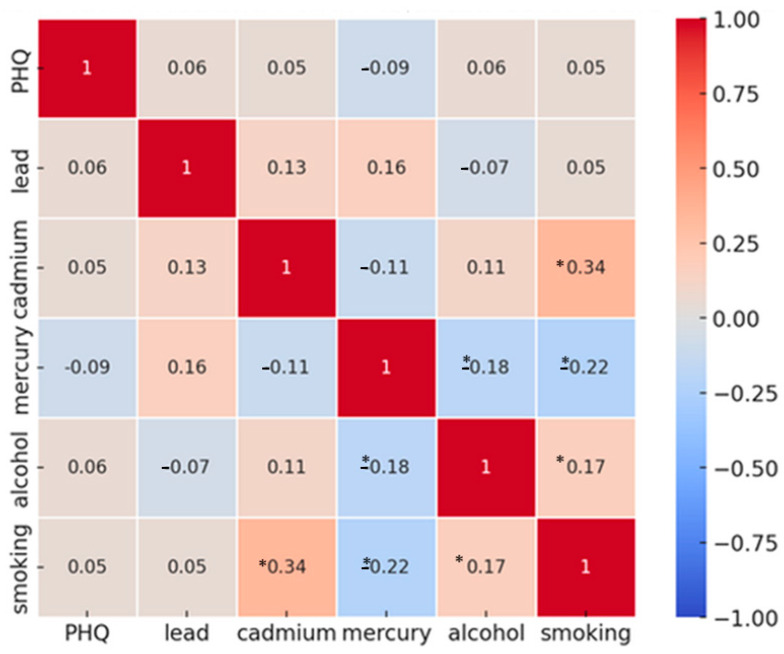
Spearman correlation among variables of interest. Symbol (*) means *p* value < 0.05.

Figure 1 displays the Spearman correlation matrix for the key study variables, including PHQ-9 depression scores, heavy metal exposure (lead, cadmium, total mercury), and behavioral factors (alcohol consumption and smoking). The depression score (PHQ-9) shows a positive correlation with lead, cadmium, alcohol, and smoking, while exhibiting a negative correlation with total mercury. Additionally, lead is positively correlated with cadmium and total mercury. Cadmium also correlates positively with smoking and negatively with alcohol, while total mercury correlates negatively with both alcohol and smoking. Alcohol consumption is positively correlated with smoking. Statistically significant values have been indicated with the asterisk (*) symbol. These correlations provide insight into the interrelationships between environmental exposure, behavioral factors, and depressive symptoms within the study population.

### 3.4. Bayesian Kernel Machine Regression Analysis

#### 3.4.1. Posterior Inclusion Probability Analysis

Table 3 presents the Bayesian Kernel Machine Regression (BKMR) results, highlighting the group and conditional posterior inclusion probabilities (PIPs) for environmental and behavioral factors associated with depressive symptoms. The environmental variables—lead, cadmium, and total mercury—are grouped together, with a group PIP of 0.511, indicating their collective importance in the model. Among these, cadmium has the highest conditional PIP (0.447), suggesting a stronger individual contribution compared to lead (0.274) and total mercury (0.280). Behavioral factors, including alcohol consumption and smoking, are grouped, with a group PIP of 0.367. Within this group, alcohol exhibits a higher conditional PIP (0.565) compared to smoking (0.435), indicating alcohol’s greater individual contribution to the depressive symptoms in the study. These PIPs offer insight into the relative importance of each factor in the context of depression.

#### 3.4.2. Univariate Exposure–Response of Metals and Behavioral Predictors for Depression

**Figure 2:**
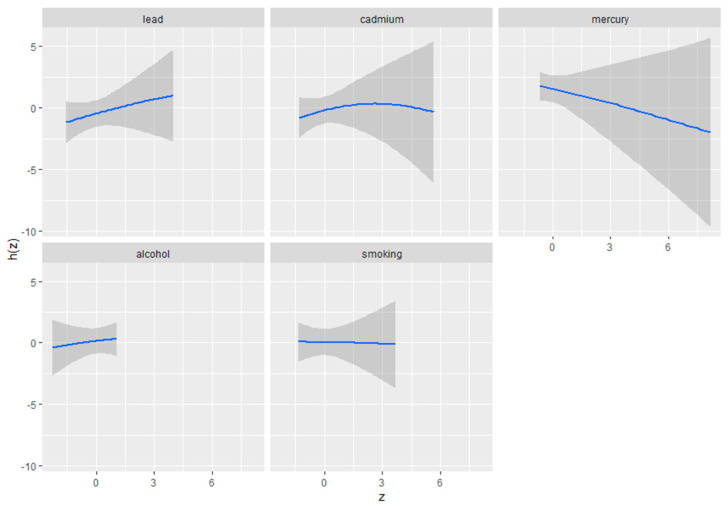
Univariate exposure–response functions and 95% credible interval for the association between single metal/behavioral exposure and depression symptoms when other metals and behavior exposures are fixed at the median. Analysis adjusted for age, sex, ethnicity, and income. Mercury labeled here is total mercury.

Figure 2 illustrates the univariate exposure–response functions and their 95% credible intervals for the association between individual metal or behavioral exposures and depressive symptoms, while holding other metals and behavioral exposures at their median values. The figure demonstrates how each single exposure—lead, cadmium, total mercury, alcohol consumption, and smoking—relates to depression when controlling for the median levels of the other variables in the model. The exposure–response functions provide insight into the direction and magnitude of the relationships between each specific exposure and depressive symptoms. The shaded areas around each curve represent the 95% credible intervals, indicating the level of uncertainty around the estimated associations. In this figure, “z” represents the value of a specific exposure, and “h(z)” represents the predicted outcome based on that exposure. Overall, this figure offers a detailed view of the impact of each environmental and behavioral exposure on depression, taking into account the influence of the other factors in the analysis.

#### 3.4.3. Bivariate Exposure–Response of Metals and Behavioral Predictors for Depression

**Figure 3:**
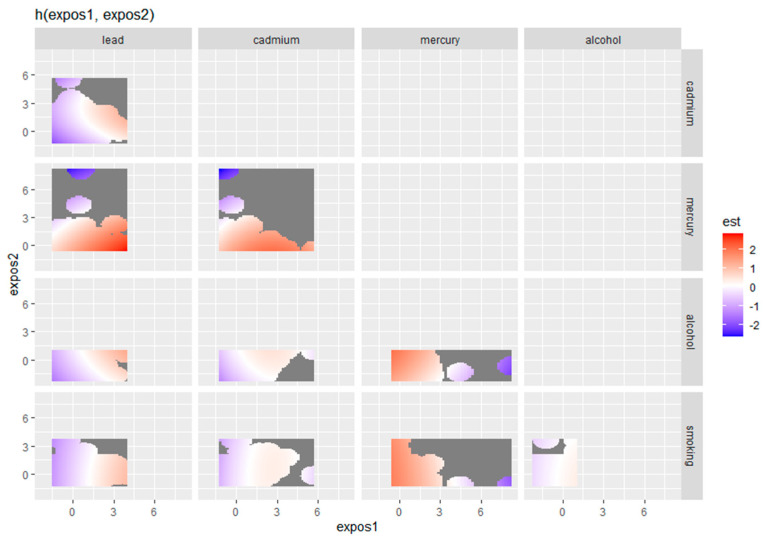
Bivariate exposure–response function of metals with depressive symptoms. Analysis adjusted for age, sex, ethnicity, and income. Mercury labeled here is total mercury.

Figure 3 displays the bivariate exposure–response functions for pairs of metals and behavioral factors in relation to depressive symptoms. In this graph, “expos1” represents the values of the first exposure (z1), and “expos2” represents the values of the second exposure (z2). These exposures include lead, cadmium, total mercury, alcohol use, or smoking, depending on the specific facet of the plot being examined. The plot illustrates the joint effects of two exposures on depression, with each panel representing a combination of two variables (e.g., lead and cadmium, total mercury and alcohol). The color scale indicates the strength and direction of the estimated associations, with red shading representing positive associations and blue shading representing negative associations. The darker gray areas indicate regions of uncertainty where the estimates are less reliable. These bivariate plots provide a detailed understanding of how pairs of environmental and behavioral exposures interact and their combined influence on depressive symptoms. 

**Figure 4:**
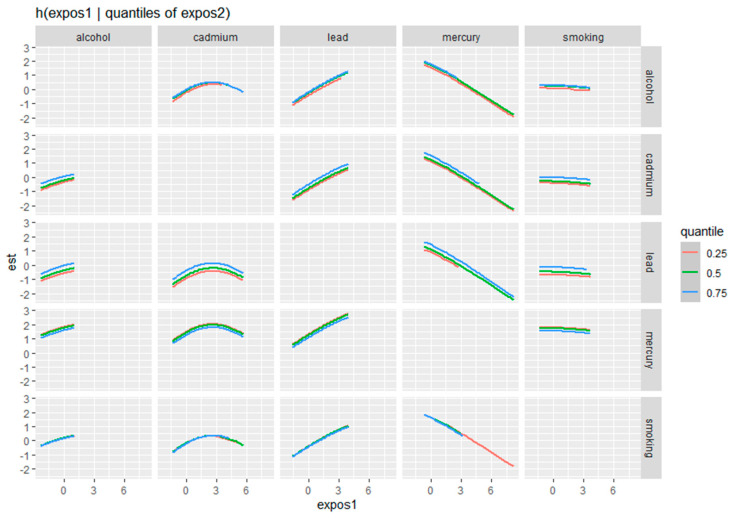
Bivariate exposure–response function of metals with depressive symptoms, investigating the predictor–response function with varying quantiles of the second predictor, while other predictors are fixed. Analysis adjusted for age, sex, ethnicity, and income. Expos 1 is the value for the first exposure, which is labeled at the top of each column in the graph, and est represents the effect of depression. The mercury labeled here is total mercury.

Figure 4 illustrates the bivariate exposure–response functions of metals and behavioral factors in relation to depressive symptoms, with the response of one exposure assessed across varying quantiles (0.25, 0.5, and 0.75) of the second exposure. Each panel represents a specific pair of variables, such as alcohol and cadmium, lead and total mercury, or smoking and lead. The curves within each panel show how the estimated effect (y-axis) of the first exposure changes as it increases (x-axis) while holding the second exposure at different quantile levels, represented by distinct color-coded lines.

Figure 4 allows for a nuanced exploration of how the relationship between one predictor and depression symptoms varies depending on different levels of a second predictor. It provides insights into potential interactions between these exposures and their combined effects on depressive outcomes. The consistent shapes and patterns across quantiles reveal whether the impact of one exposure strengthens, weakens, or remains stable as the second predictor changes. In Figure 4, “expos1” denotes the values of the first exposure, as labeled at the top of each column (e.g., lead, cadmium, total mercury, alcohol use, or smoking), while “est” represents the estimated effect of the exposure on depression as modeled by BKMR.

#### 3.4.4. Overall Effect Summary of Metals and Behavioral Predictors on Depression

**Figure 5:**
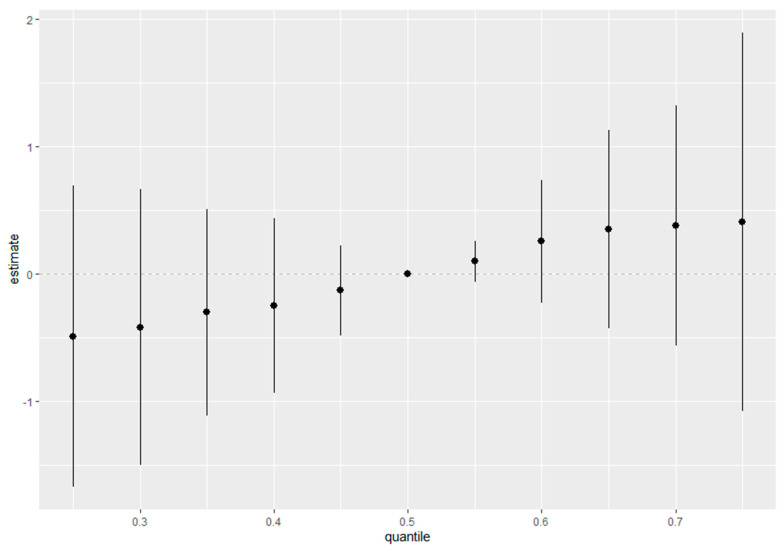
Summary of overall health effects of exposures on depressive symptoms explored at various quantiles (from 25th to 75th percentiles). Analysis adjusted for age, sex, ethnicity, and income.

Figure 5 presents a summary of the overall health effects of various exposures on depressive symptoms, analyzed across different quantiles, ranging from the 25th to 75th percentiles as compared to the 50th percentile. The y-axis represents the estimated effect, while the x-axis shows the quantiles of exposure. Each point in the plot represents the estimate at a specific quantile, with the vertical lines indicating the 95% credible intervals around these estimates. The figure demonstrates how the estimated health effects of these exposures on depression vary across the quantiles, providing insights into whether the relationship between exposures and depressive symptoms changes with increasing levels of exposure. This visualization aids in understanding whether certain quantiles of exposure have stronger or weaker associations with depressive outcomes. The trend and credible intervals at each quantile allow for a comprehensive exploration of how the effects of the exposures evolve across the distribution. In Figure 5, the x-axis (“quantile”) represents the quantiles of all predictors fixed at specific percentiles (e.g., 25th to 75th), while the y-axis (“estimate”) indicates the overall estimated effect of the predictors on the outcome (depression) relative to the reference level (50th percentile).

#### 3.4.5. Single-Exposure Effect Analysis 

**Figure 6:**
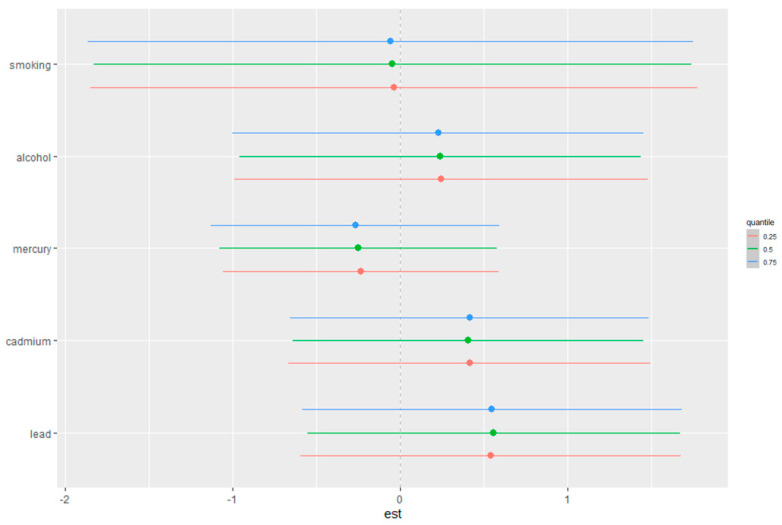
Single-exposure AL effects (95% CI), defined as the change in the response associated with a change in a particular exposure from its 25th to its 75th percentile, where all of the other exposures are fixed at a specific quantile (0.25, 0.50, or 0.75). Analysis adjusted for age, sex, ethnicity, and income. Mercury labeled here is total mercury.

Figure 6 illustrates the single-exposure effects on depressive symptoms, showing the estimated changes in the response when a particular exposure shifts from its 25th to its 75th percentile, with all other exposures are held constant at different quantiles (0.25, 0.50, or 0.75). The exposures included are smoking, alcohol consumption, total mercury, cadmium, and lead. The horizontal lines represent the 95% credible intervals for each estimate, with different colored points corresponding to the three quantiles (blue for 0.25, green for 0.50, and red for 0.75).

The plot provides a comparison of how the effect of each exposure on depressive symptoms varies depending on the quantile at which the other exposures are held constant. For example, the effect of lead exposure appears to slightly increase across quantiles, while the effects of alcohol and smoking show minimal variation across the different quantile levels. This figure offers an in-depth examination of how individual exposures interact with depressive symptoms across different levels of other exposures, helping to highlight potential thresholds or non-linear effects within the study population. In Figure 6, the x-axis labeled “est” represents the estimated change in the outcome when each predictor changes from its 25th percentile to its 75th percentile, while the other predictors are held constant at specific fixed quantiles (e.g., 25th, 50th, or 75th percentile).

**Figure 7:**
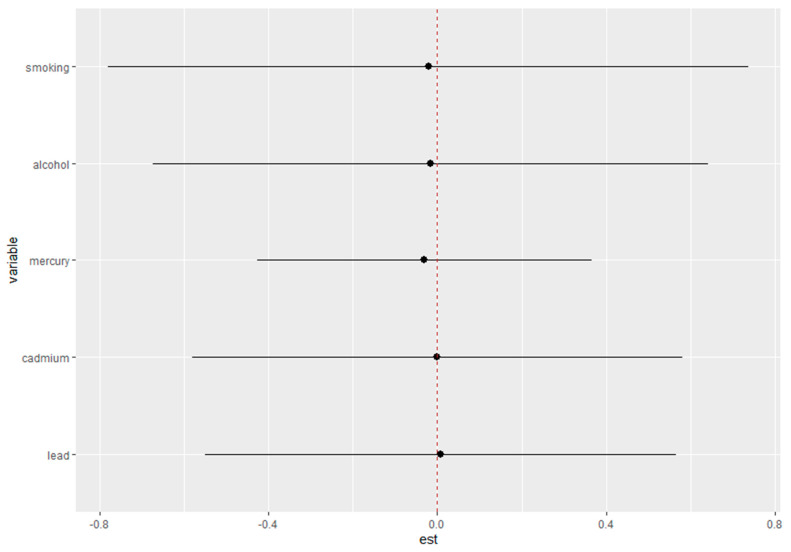
Single-variable interaction terms for total mercury, cadmium, lead, alcohol consumption, and smoking. Analysis adjusted for age, sex, ethnicity, and income.

Figure 7 presents the single-variable interaction terms for total mercury, cadmium, lead, alcohol consumption, and smoking in relation to depressive symptoms. The estimated effects (est) for each variable are plotted along the x-axis, with the corresponding 95% credible intervals represented by horizontal lines. The vertical red dashed line at zero indicates the null value, where no effect is present. The black dots represent the point estimates for each interaction term.

This figure explores how each individual exposure interacts with depressive symptoms when considered independently. The credible intervals for each variable provide insights into the variability of these interactions and whether the effects are likely to differ from zero. The visual representation underscores the estimated effect size for each variable, helping to identify potential significant contributors to depressive symptoms based on their interaction terms. In Figure 7, the x-axis labeled “est” quantifies the change in single-predictor depression risks when the other predictors are fixed at their 75th percentile compared to their 25th percentile, highlighting the interaction effects between exposures on the outcome.

## 4. Discussion

This study aimed to investigate the combined effects of environmental pollutants (lead, cadmium, total mercury) and behavioral factors (alcohol consumption, smoking) on depressive symptoms in women. This is critical as women experience a higher rate of depression than men. 

The results from the Spearman correlation analysis and linear regression highlight important but limited insights into the relationships between depressive symptoms and environmental/behavioral exposures. As shown in the correlation plot (Figure 1), significant associations (denoted by asterisks) were observed between certain variables, such as smoking and cadmium (r = 0.34, *p* < 0.05) and alcohol and smoking (r = 0.17, *p* < 0.05). However, many of the observed correlations were weak and nonsignificant, as were the regression coefficients in Table 2. This suggests that traditional methods like Spearman correlation and linear regression may not fully capture the complex, non-linear, and interactive relationships between predictors and depressive symptoms. While these methods provide insights into the direction and magnitude of individual associations, they are inherently limited in their ability to account for the combined and cumulative effects of multiple exposures.

This limitation underscores the need for more advanced statistical techniques like BKMR. BKMR goes beyond simple pairwise correlations and linear associations by modeling exposure–response relationships nonparametrically. It accounts for the interactions and potential synergistic effects between exposures while allowing for non-linear relationships with the outcome. For example, while linear regression suggested nonsignificant effects for most exposures, BKMR identified cadmium and alcohol as significant contributors to depressive symptoms through their posterior inclusion probabilities. Moreover, BKMR’s exposure–response curves revealed patterns—such as the non-linear and complex effects of cadmium—that were not evident in the linear models.

Our study found that behavioral factors, particularly alcohol, significantly influenced depressive symptoms, with combined environmental and behavioral exposures amplifying their impact. This underscores the need for an integrated approach addressing both environmental and lifestyle factors in women. Previous research supports a positive relationship between alcohol consumption and depression, as shown in both clinical and population-based studies [33]. Some studies indicate that women are more likely to use alcohol to cope with psychological stress, and those with alcohol use disorder are more likely also to experience depression [34]. Research from the COVID-19 pandemic indicates that women were more susceptible to psychological distress than men, which increased their likelihood of using alcohol to cope with pandemic-related stress [35]. In this context, heavy alcohol use was linked to high levels of COVID-19-related distress in women but not in men [36], with alcohol consumption in women rising by 17% during lockdown [37]. A number of studies have also found that the relationship between alcohol use disorders and major depression is stronger for women than for men [38,39]. However, sex differences in the relationship between alcohol consumption and depression are not so clear, with some studies finding a stronger relationship for females for usual quantity [40] and heavy episodic drinking but others finding a stronger relationship between recent symptoms of depression and a hazardous/harmful level of drinking for men than for women [41]. 

This study revealed that cadmium is a strong contributing factor to depression among women. It is worth noting that many studies confirm that women are particularly at risk of developing depression at any time in their lives [42,43]. A study examining the link between blood cadmium levels and depression in adult women in the U.S. [44] suggested that cadmium exposure may disrupt the monoaminergic neurotransmitter system, which is involved in regulating mood, emotions, and cognitive functions, potentially contributing to the onset of depression. Cadmium, commonly encountered in daily life, enters the body through activities such as smoking, welding, skin contact, and exposure to cadmium-containing products such as food [45,46]. It accumulates in the central nervous system, affecting neurotransmitter levels (serotonin, dopamine, and norepinephrine), which may contribute to the development of depression [47]. 

In the case of lead and total mercury, the findings from this study suggest that lead is significantly associated with depression, while increased exposure to total mercury is associated with fewer depressive symptoms. In a study by Kamińska and colleagues, there was no statistically significant correlation between blood lead levels and depressive symptoms in women [48]. Meanwhile, previous research has shown that exposure to lead may be a factor in the development of depressive symptoms in menopausal women [49]. Regarding the paradoxical results associated with total mercury in our study, researchers have found that the omega-3 fatty acids present in fish may mitigate the impact of methymercury on depression [50]. Thus, the protective effects observed in this study are likely due to the beneficial influence of omega-3 fatty acids, which mitigate the harmful effects of methylmercury exposure 

Based on this investigation, the effect of smoking on depressive symptoms in women is relatively lower compared to alcohol use. This contrasts with most studies linking smoking to higher stress and mental issues [51]. Some studies have found a stronger relationship between smoking and depression in females compared to males. Other studies reported a gender-specific effect, indicating that the smoking–depression link is significantly greater in females than in males, particularly among adolescents [52] and adults [53]. Also, a study on the epidemiology of depressive disorder [54] revealed that women had a higher prevalence of depression than men. When smoking and alcohol consumption occur together, as they often do, their combined impact on depression can be more severe. Both substances can disrupt mood-regulating neurotransmitters, worsen symptoms of depression, and create difficulties in withdrawal and recovery [55]. 

This present study highlights the critical role of cumulative exposure to environmental and behavioral stressors in women’s mental health. Combined exposure to heavy metals and behaviors like smoking and drinking amplifies the risk of depression, as environmental toxics disrupt hormonal and neurotransmitter systems, while risky behaviors further alter brain chemistry. Simultaneous exposures can overwhelm mental health defenses, significantly increasing the susceptibility to depression. Living in polluted environments exposes individuals to harmful substances (e.g., heavy metals, particulate matter, chemicals) that can affect both physical and mental health. For example, smoking can worsen respiratory problems caused by pollution, leading to increased physical discomfort and contributing to depressive symptoms. 

Alcohol, cadmium, and lead showed strong associations with depression. Cadmium exhibited a non-linear effect, with depressive symptoms sharply increasing at higher exposure levels before slightly declining, suggesting a potential threshold where the body’s defenses may be temporarily overwhelmed (Figure 4). These exposures can disrupt the hypothalamic–pituitary–adrenal (HPA) axis, leading to stress dysregulation, inflammation, and immune dysfunction, contributing to depression by impairing brain function and mood regulation [56]. As a heavy metal, cadmium disrupts mitochondrial function, reduces ATP production, and increases oxidative stress, contributing to hormonal imbalances and mood disorders like depression and anxiety [47]. Mitochondria are essential for energy production, and their dysfunction can cause cellular damage and death, which may affect the brain regions involved in mood regulation. Cadmium can mimic or interfere with the action of hormones by binding to hormone receptors, such as estrogen receptors (ER-α and ER-β), and activating their signaling pathways inappropriately, even in the absence of natural hormones [57,58]. Additionally, cadmium can disrupt the synthesis and metabolism of endogenous hormones, alter hormone-regulated gene expression, and compete with essential metals like zinc, which is critical for proper hormonal receptor function. These disruptions can lead to significant imbalances in estrogen and testosterone levels, ultimately affecting hormonal homeostasis [59,60,61]. Hormonal imbalances are known to influence mood, and disturbances in these hormones can contribute to the development of depression and anxiety.

Cumulative exposures to environmental stressors, such as pollutants, heavy metals, and lifestyle factors, can significantly disrupt the body’s stress response systems. Prolonged exposure to these stressors can result in persistently elevated cortisol levels, which are most commonly measured in serum or saliva for acute effects, with hair cortisol levels often used to assess chronic stress-related disruptions [62]. Chronic high cortisol is linked to various health issues, including anxiety and depression, as it can impair brain function, reduce neurogenesis, and disrupt mood-regulating neurotransmitters [63]. Individuals exposed to chronic stress may resort to unhealthy coping mechanisms, such as substance abuse, overeating, or social withdrawal, which can worsen mental health outcomes. The allostatic load/overload model posits that the cumulative effects of multiple stressors can overwhelm the body’s regulatory systems, leading to a decline in mental health [64]. Allostasis refers to the body’s ability to adapt to stressors and maintain stability through change. While this adaptive response is crucial for survival, chronic exposure to stressors can result in allostatic load [65], which refers to the cumulative physiological burden on the body caused by the repeated activation of stress response systems. This wear and tear involves the dysregulation of systems such as the hypothalamic–pituitary–adrenal (HPA) axis, immune function, and cardiovascular regulation, leading to an increased risk of various chronic diseases, including mental health disorders, cardiovascular disease, and metabolic syndromes.

Addressing depressive symptoms and stress-related conditions requires a holistic public health approach that integrates environmental and behavioral factors. Exposure to pollutants like cadmium, lead, and air pollution is linked to mental health issues, while risky behaviors such as smoking and poor diets can amplify these effects. Strategies should prioritize reducing exposures through stricter regulations, cleaner technologies, and community awareness, while promoting healthy behaviors like physical activity and nutrition to build resilience. Vulnerable populations, including low-income and marginalized groups, face disproportionate risks, highlighting the need for integrated policies that combine environmental health, behavioral interventions, and social support to address cumulative health impacts effectively. 

### 4.1. Limitations

The reliance on cross-sectional data in this study presents a limitation, as it only captures a snapshot in time, highlighting associations instead of establishing causal relationships. Without tracking changes over time, it is challenging to determine if an exposure causes an outcome or if the association is affected by other variables, limiting conclusions about directionality. Additionally, measurement issues, such as biases in self-reported behaviors like alcohol and smoking or inaccuracies in environmental exposure assessments, can skew results. Inaccuracies in exposure measurements, such as those for environmental toxics like cadmium or lead, may occur due to limitations in detection methods, variations in sampling procedures, or fluctuations in exposure levels over time. Unmeasured factors, including genetic predispositions and psychosocial stressors, may also impact these associations, making it crucial to account for a broad range of confounders for more reliable findings.

### 4.2. Future Research

Future research should emphasize longitudinal studies to gain a clearer understanding of how cumulative environmental and behavioral exposures affect depression over time. By tracking individuals over extended periods, researchers could observe mental health changes in response to persistent or varying exposures, helping to reveal potential causal pathways. This approach would offer insights into how combined stressors influence depressive symptoms and inform more effective prevention and intervention strategies. Exposure limits for lead (≥5 μg/dL in blood), total mercury (≥50 μg/L in urine), and indoor carbon monoxide (CO) levels have been defined to guide health interventions. The World Health Organization (WHO) recommends CO exposure thresholds of 9–10 ppm for no more than 8 h, 25–35 ppm for no more than 1 h, and 90–100 ppm for no more than 15 min [66]. However, additional research is necessary to establish clear exposure thresholds for lead, total mercury, and smoking that specifically heighten the risk of depressive symptoms. Defining these thresholds would improve our understanding of when these exposures pose significant mental health risks, refine public health recommendations, strengthen regulatory guidelines, and enable targeted interventions to mitigate the risks associated with these substances. 

Utilizing advanced modeling techniques, such as machine learning, can significantly improve our understanding of how multiple exposures and stressors collectively impact mental health risks. These techniques allow for an in-depth analysis of complex interactions between factors like environmental toxics, lifestyle choices, and psychosocial stressors, which traditional statistical methods may not capture as effectively. Machine learning models can uncover intricate patterns and relationships among these variables, revealing additional risk factors for mental health issues, including depression. By offering a more detailed view of these interactions, machine learning can enhance risk prediction and inform more tailored mental health interventions.

## 5. Conclusions

In conclusion, it is essential to consider both the individual and cumulative impacts of environmental and behavioral factors on depression in women. While each individual factor can uniquely contribute to depressive symptoms, their interactions may create compounded effects that greatly influence overall well-being. By analyzing both individual exposures and their cumulative interactions, researchers and policymakers can achieve a more thorough understanding of the mechanisms that contribute to depressive symptoms. This comprehensive perspective is essential for identifying not just vulnerable3 women but also vulnerable populations, formulating effective interventions, and crafting public health strategies that address the complex nature of depression, ultimately leading to improved mental health outcomes. Also, there is an urgent need for integrated public health strategies and additional research focused on reducing the combined effects of multiple stressors on mental health. As the interactions between environmental, behavioral, and social factors become clearer, it is essential to adopt a collaborative approach that includes various sectors such as health, environment, and community development. By bringing together researchers, policymakers, and public health professionals, we can create comprehensive interventions that address the complex nature of mental health issues. Furthermore, continued research is vital to enhance our understanding of how different stressors interact and influence mental health outcomes, allowing us to identify effective prevention strategies and optimize resource allocation. Ultimately, a coordinated effort is necessary to alleviate the impact of mental health challenges in our communities and to foster overall well-being.

## Figures and Tables

**Table 1:** Descriptive statistics for study continuous variables.

Variable	N	Mean	Std. Dev.
PHQ	153	5.94	5.73
Lead (µg/dL)	153	1.21	0.679
Cadmium (µg/L)	153	1.47	1.06
Total mercury (µg/L)	153	0.800	0.932
Age (Years)	153	48.0	14.9

Note PHQ = PHQ-9, a measure of depressive symptoms.

**Table 2:** Linear regression results for depression.

Variable	* Coef.	Std. Err.	*p*-Value	95% Conf. Interval
Lead	1.21	0.820	0.140	−0.405, 2.83
Cadmium	0.198	0.474	0.677	−0.739, 1.13
Total mercury	−0.520	0.532	0.331	−1.57, 0.533
Alcohol	0.137	0.165	0.408	−0.189, 0.463
Smoking	0.010	0.067	0.877	−0.122, 0.143

* Adjusted for age, sex, ethnicity, and income.

**Table 3:** BKMR analysis of depressive symptoms: group and conditional posterior inclusion probabilities for environmental and behavioral factors.

Variable	Group	Group PIP	Cond PIP
Lead	1	0.511	0.274
Cadmium	1	0.511	0.447
Total mercury	1	0.511	0.280
Alcohol	2	0.367	0.565
Smoking	2	0.367	0.435

## Data Availability

The data presented in this study are openly available on the CDC NHANES site at https://wwwn.cdc.gov/nchs/nhanes/continuousnhanes/overview.aspx?BeginYear=2017 (accessed on 1 December 2024).
